# Hybrid Polymer–Surfactant Wormlike Micelles for Concurrent Use for Oil Recovery and Drag Reduction

**DOI:** 10.3390/polym15234615

**Published:** 2023-12-04

**Authors:** Alexander L. Kwiatkowski, Vyacheslav S. Molchanov, Yuri M. Chesnokov, Oleksandr I. Ivankov, Olga E. Philippova

**Affiliations:** 1 Faculty of Physics, Lomonosov Moscow State University, 119991 Moscow, Russia; molchan@polly.phys.msu.ru (V.S.M.); phil@polly.phys.msu.ru (O.E.P.); 2 National Research Center, Kurchatov Institute, 123182 Moscow, Russia; chessyura@yandex.ru; 3 Joint Institute for Nuclear Research, 141980 Dubna, Russia; ivankov@jinr.ru

**Keywords:** hybrid micelles, wormlike surfactant micelles, hydrocarbon, hydraulic fracturing, drag reduction

## Abstract

We report on the effect of a hydrocarbon (n-dodecane) on the rheological properties and shapes of the hybrid wormlike micelles (WLMs) of a surfactant potassium oleate with an embedded polymer poly(4-vinylpyridine). With and without hydrocarbon solutions, the hybrid micelles exhibit the same values of viscosity at shear rates typical for hydraulic fracturing (HF) tests, as solutions of polymer-free WLMs. Therefore, similar to WLMs of surfactants, they could be applied as thickeners in HF fluids without breakers. At the same time, in the presence of n-dodecane, the hybrid micelles have much higher drag-reducing efficiency compared to microemulsions formed in polymer-free systems since they form “beads-on-string” structures according to results obtained using cryo-transmission electron microscopy (cryo-TEM), dynamic-light scattering (DLS), and small-angle X-ray scattering (SAXS). Consequently, they could also act as drag-reducing agents in the pipeline transport of recovered oil. Such a unique multi-functional additive to a fracturing fluid, which permits its concurrent use in oil production and oil transportation, has not been proposed before.

## 1. Introduction

The self-assembly of amphiphilic molecules of surfactants in aqueous media results in the formation of micelles of various geometries [1,2,3,4,5,6,7,8]. Of particular interest are wormlike micelles (WLMs), that represent semi-flexible cylindrical aggregates reaching up to several micrometers in length [9,10,11,12,13]. The rheological properties of semi-dilute solutions of WLMs are similar to those of polymers [14,15]. At low shear stress, their viscosity can exceed 1000 Pa·s, which is due to the presence of a physical network of entangled chains [9,10,16]. But at high shear stress, their viscosity decreases dramatically (to 1–100 mPa·s), as the chains are disengaged from the entanglements and aligned along the direction of flow. Solutions of WLMs and polymers play an important role in the oil industry, where they are applied as thickeners in hydraulic fracturing (HF) [10,17,18,19,20,21,22] and as drag-reducing agents (DRAs) in oil transportation [10,17,23,24,25].

HF is a widely used method for enhanced oil recovery in which oil is collected not only from the oil well but also from the surrounding artificial fractures in oil-bearing rock [19]. The fractures are filled with a HF fluid containing proppant particles to keep the fractures open while producing oil. Stuck proppants prevent the squeezing of the fracture. A HF fluid should have high viscoelasticity to prevent proppants from undergoing sedimentation. Toward this aim, semi-dilute solutions of WLMs and polymers are commonly used as thickeners [17,18,19,20,21]. When returning a well to production, the viscoelasticity has to be reduced to provide access of the oil from a fracture to the well. To achieve this aim, in the case of polymer-based HF fluids, special compounds called breakers (e.g., ammonium persulfate) are added to break the strong covalent bonds in polymer chains [26]. Unlike polymers, the WLMs are formed through weak non-covalent hydrophobic interactions [2], which endow WLMs with high sensitivity to hydrophobic additives [21,27,28]. In particular, the solubilization of hydrocarbons by the WLMs of a surfactant induces their gradual transformation into microemulsion droplets [29]. For this reason, the viscosity of micellar solutions is spontaneously diminished up to the viscosity of water. This effect allows surfactant-based HF fluids to be used without breakers.

Recovered oil has to be transported to consumers. During the transportation of oil in pipeline networks, drag or frictional losses appreciably affect flowing capacity and pumping rates [30]. Compensation of the losses requires supplementary energy costs, resulting in a subsequent drop in marginality. One of possible ways of avoiding this is to use DRAs to decrease the turbulence of the flow and, consequently, reduce drag on the pipe walls [23]. For this application, polymers are employed [10]. Under the same conditions, the drag-reducing effect of WLMs is much lower since WLMs, unlike polymers, transform into microemulsion droplets in the presence of hydrocarbons [29]. 

A promising system combining the advantages of polymer-based and surfactant-based fluids constitutes hybrid WLMs of surfactants with embedded polymer chains [31]. Such a system will retain the responsiveness to hydrocarbons and the anti-degradation ability inherent to surfactant-based fluids and, at the same time, exhibit the high drag reducing efficiency inherent to polymer-based fluids. These properties will allow WLMs armored with polymer chains to be sequentially applied for HF and drag reduction. The hybrid WLMs of the anionic surfactant potassium oleate with the embedded non-ionic polymer poly(4-vinylpyridine) (P4VP) reported recently [31,32] can serve as an example of such a nanomaterial. Rheological experiments showed the high viscoelasticity of the semi-dilute solutions of the complexes due to the formation of a physical network of entanglements, as evidenced via cryo-transmission electron microscopy (cryo-TEM) [31]. In this paper, the influence of the hydrocarbon n-dodecane on the rheological properties and shape of this system is investigated. We demonstrate that the hybrid P4VP–potassium oleate WLMs represent a unique multi-functional additive to fracturing fluid that permits its application both in oil production and transportation.

## 2. Materials and Methods

### 2.1. Materials

The surfactant potassium oleate (TCI Europe, >98% purity), the inorganic salt potassium chloride (Fluka, >99.5% purity, Charlotte, NC, USA), the polymer poly(4-vinylpyridine) (P4VP) (Aldrich, >99% purity, St. Louis, MO, USA), ethanol (Merck, >99% purity, Rahway, NJ, USA), and the hydrocarbon n-dodecane (Fluka, >99% purity) were used as received without further purification. The value of the molecular weight of P4VP was equal to 228,000 g/mol, as previously determined via static light scattering in an ethanol solution [31]. An aqueous solution of 1M of potassium hydroxide (Merck, 85% purity) was used to adjust pH in all solutions to 11. Water was purified using a Millipore Milli-Q system (Merck Millipore, Burlington, NJ, USA).

### 2.2. Samples Preparation

Stock solution of WLMs of potassium oleate was prepared by mixing potassium oleate and potassium chloride in Milli-Q water with a magnetic stirrer for 1 day. The concentrations of potassium oleate and KCl equaled 1.5 and 6 wt%, respectively.

Hybrid P4VP-potassium oleate micelles were prepared via solubilization of a thin film of P4VP, preliminarily obtained at the bottom of a vial, in stock solution of WLMs. To prepare the thin film of P4VP, an appropriate amount of a 5 wt% solution of P4VP in ethanol was poured into the vial and left at room temperature until the full evaporation of ethanol has occurred. Concentration of polymer in the solutions was equal to 0.2 wt%, which was previously shown to correspond to polymer–surfactant micelles saturated with P4VP [32]. Solutions of hybrid micelles were mixed using magnetic stirrer for 1 day.

To study the effect of hydrocarbon, 1 wt% dodecane was added to solutions of WLMs without the polymer or to solutions of hybrid P4VP-potassium oleate micelles, stirred for 1 day, and left to equilibrate for another day.

### 2.3. Rheology

Rheological measurements were performed using a controlled-stress rheometer, Anton Paar Physica MCR 301 (Ostfildern, Germany), at 20 °C. Cone–plate (diameter of 50 mm; cone angle of 1°) and coaxial cylinder (inner diameter of 24.661 mm; outer diameter of 26.667 mm) measuring cells were used for the experiments with samples of high and low viscosity, respectively. Steady shear experiments were carried out in the range of shear rates from 4·10^−3^ to 3·10^3^ s^−1^. Frequency sweep experiments were carried out in the linear viscoelastic range at angular frequencies ω from 0.07 to 90 rad/s.

First, normal stress difference *N*_1_ measurements were performed using a controlled-stress rheometer Anton Paar MCR 102e (Anton Paar GmbH, Graz, Austria) equipped with normal force sensor, using a measuring cell cone–plate (diameter 50 mm, cone angle 1°) at shear rates varying from 10 to 3000 s^−1^ at 20 °C. As proposed by C.W. Macosco [33], the correction inertia term was added to the rheometer output value of *N*_1_. The real value of *N*_1_ in the cone–plate geometry was estimated from the normal force *F_N_*, detected using the rheometer’s sensor:

$$N_{1}=\frac{2F_{N}}{\pi R^{2}}+0.15\;\rho \;\Omega^{2}R^{2},$$

where *R* is the radius of the cone, Ω is angular velocity, and *ρ* is the density of the solution, taken to be equal to 1 g/cm^3^ for all studied samples.

### 2.4. Cryogenic Transmission Electron Microscopy

Cryo-TEM study was performed using a Titan Krios (Thermo Fisher Scientific, Hillsboro, OR, USA) at 300 kV equipped with a Falcon 2 direct electron detector (Thermo Fisher Scientific, Hillsboro, OR, USA). Cryo-TEM images were acquired using EPU 3.6 software (Thermo Fisher Scientific, Hillsboro, OR, USA) at 37,000× magnification, defocus range of 3–4 µm, and total dose of 50 e/Å^2^.

Cryo-TEM specimens in native conditions were prepared using a Vitrobot Mark 4 (Thermo Fisher Scientific, Hillsboro, OR, USA) at 20 °C and 100% humidity. A total of 3 µL of the sample was deposited onto a Lacey TEM grid (Ted Pella, Northport, NY, USA) glow-discharged for 20 s at 0.26 mbar and 20 mA using Pelco EasiGlow (Ted Pella, Northport, NY, USA). The excess solution was blotted with filter paper for 3 s on both sides; after that, the grid was immediately plunged into liquid ethane.

### 2.5. Dynamic Light Scattering

ALV/DLS/SLS-5022F (ALV GmbH, Langen, Germany) goniometer system was employed for DLS measurements. It was equipped with ALV6010/EPP (ALV GmbH, Langen, Germany) digital correlator and a stepping-motor-driven variable-angle detection system. A vertically polarized helium–neon laser, operating at wavelength of 632.8 nm, was used as a light source. Temperature was kept at 20 °C using Lauda Ecoline RE 306 system (Lauda, Lauda-Königshofen, Germany). Before the measurements were taken, the solutions were filtered through 0.45 μm Millipore Millex-FG filters (Merck Millipore, Burlington, NJ, USA) to prevent exposure to dust. Details of the experiments and data treatment are described elsewhere [34].

### 2.6. Small-Angle X-ray Scattering

SAXS measurements were carried out using the Xeuss 3.0 SAXS/WAXS System (JINR, Dubna, Russia) via a GeniX3D microfocus generator of X-ray radiation with CuKα (λ = 0.1541889 nm) operating in the 30 W/30 µm mode. The spectrometer was equipped with a moving detector, Eiger 2R 1M 2D-detector (Dectris, Baden, Switzerland), with a sensitive area of 77.1 × 79.7 mm^2^ and integrated with the XSACT program. The measurements were carried out using three different positions of detectors (Eiger2 R 1M 2D-detector (Dectris, Baden, Switzerland), with a sensitive area of 77.1 × 79.7 mm^2^): 350, 1800, and 4500 mm. Data treatment was conducted using XSACT program, enabling the determination of the X-ray-scattering intensity *I(Q)* in the range of momentum transfer of 0.007 < *Q* < 0.7 Å^−1^. The measurements were carried out in a vacuum at room temperature.

To obtain the radii of the microemulsion droplets in an aqueous solution of potassium oleate with added n-dodecane, the fitting of the scattering curves *I*(*Q*) in absolute units using the model of a spherical particle with a core–shell structure was performed using SASview-5.0 software [35]. The scattering intensity *I*(*Q*) of the spheres with the radius of the core *r_c_* and thickness of the shell *r_s_* is given by the following expression [36]:

$$P(Q)={\left(\frac{3}{V_{}}\left[V_{c}\left(\rho_{c}-\rho_{s}\right)\frac{\sin(Qr_{c})-Qr_{c}\cos(Qr_{c})}{{\left(Qr_{c}\right)}^{3}}+V\left(\rho_{s}-\rho_{solvent}\right)\frac{\sin(Qr)-Qr_{c}\cos(Qr)}{{\left(Qr\right)}^{3}}\right]\right)}^{2},$$

where *r* = *r_c_* + *r_s_* is the radius of the whole particle, $V=\frac{4\pi {\left(r_{c}+r_{s}\right)}^{3}}{3}$ is the volume of the particle, $V_{c}=\frac{4\pi r_{c}^{3}}{3}$ is the volume of the core, *ρ_c_* = 7.3·10^−6^ Å^−2^ is the X-ray scattering length density of the core of n-dodecane, *ρ_s_* = 9.8·10^−6^ Å^−2^ is the X-ray scattering length density of the shell of potassium oleate, and *ρ_solvent_* = 9.4·10^−6^ Å^−2^ is the scattering length density of water.

## 3. Results and Discussion

**Figure 1:**
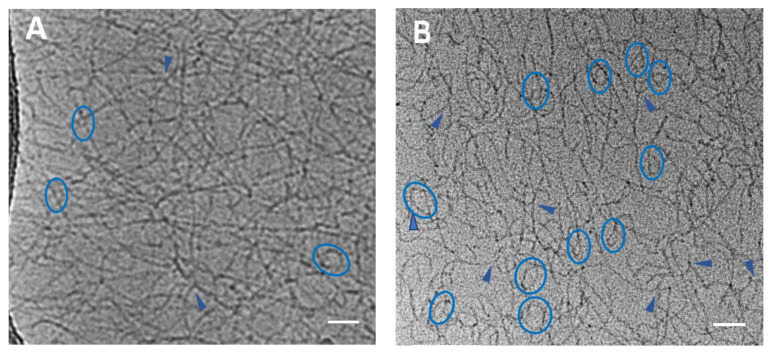

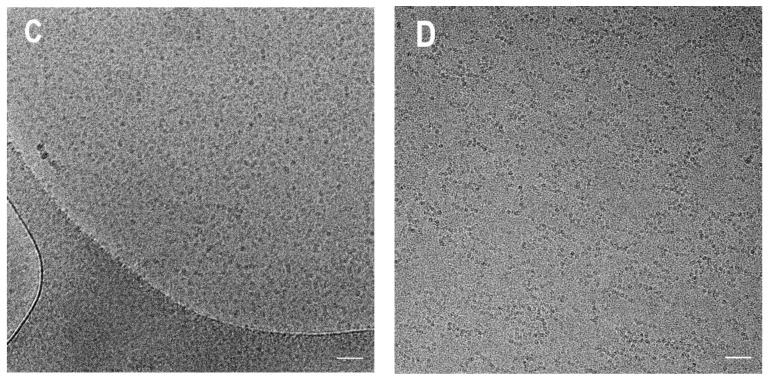
Cryo-TEM images of WLMs in 1.5 wt% aqueous solutions of potassium oleate without additives (**A**); 1.5 wt% aqueous solutions of potassium oleate saturated with 0.2 wt% of P4VP (**B**); 1.5 wt% aqueous solutions of potassium oleate with 1 wt% n-dodecane (**C**); 1.5 wt% aqueous solutions of potassium oleate saturated with 0.2 wt% of P4VP upon addition of 1 wt % n-dodecane (**D**) at pH 11. Concentration of KCl: 6 wt%. Arrows and circles denote loops and branches, respectively. Scale bars represent 50 nm.

Figure 1 shows that upon the absorption of the polymer, the micelles remain wormlike. But in contrast to polymer-free micelles, they contain many more branching points and loops (Figure 1A,B). These peculiarities of the structure of hybrid potassium oleate-P4VP micelles have already been demonstrated, specifically via computer modeling [31]. The shortening of the WLMs after the solubilization of the polymer can be explained by the increased probability that the WLMs would break at the boundaries of the polymer-containing sections since the absorbed polymer stabilizes the semi-spherical end-caps of the micelles [32]. The branching observed was attributed to the linking of the adjacent micelles by the absorbed hydrophobic polymer, permitting the minimization of the thermodynamically unfavorable contact of the polymer with water [31].

**Figure 2:**
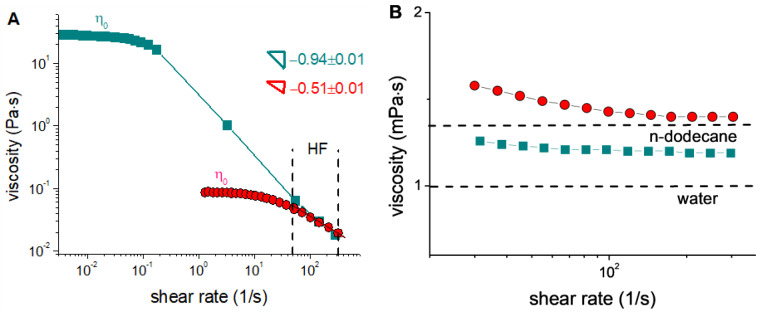
Flow curves for 1.5 wt% aqueous solutions of potassium oleate without polymer (squares) and saturated with 0.2 wt% P4VP (circles) in the absence of hydrocarbon (**A**) and in the presence of 1 wt% n-dodecane (**B**) at pH of 11. Concentration of KCl: 6 wt%. Slopes of the shear-thinning regions are indicated in the Figure. Vertical dashed lines (**A**) limit the range of the shear rates that are typical for in situ HF tests. Horizontal dashed lines (**B**) correspond to the viscosity of n-dodecane and water.

Figure 2A shows that the zero-shear viscosity *η_0_* of the solution of hybrid micelles is more than two orders of magnitude lower than that for the neat micelles, which may be due to the shortening of the WLMs and the formation of branching points and loops upon the absorption of the polymer. At the same time, the drop in viscosity with shear rate is less pronounced in the hybrid micelles: the slope of the shear-thinning region for them is −0.51 as opposed to that of −0.94 for the neat micelles. According to the literature [37,38], this can be related to the branching in the hybrid micelles, which hinders their alignment along the flow direction [39,40]. As a result, the flow curves of both the polymer-free and polymer-loaded micelles almost coincide at the higher shear rates (Figure 2A) that are typical for in situ slickwater fracturing tests of proppant transport [18,20]. Therefore, the solutions of hybrid WLMs of potassium oleate saturated with P4VP can be applied as a surfactant-based HF fluid, similar to the WLMs of potassium oleate without an added polymer.

Surfactant-based HF fluids do not require the use of breakers in order to reduce the viscosity and provide the access of the recovered oil to the well, because the absorption of hydrocarbons induces the disruption of WLMs and their transformation into spherical microemulsion droplets [10,19,29]. Such droplets almost do not contribute to the viscosity of the solution, so that it becomes close to that of water [29]. The cryo-TEM data show (Figure 1C) that such droplets were indeed formed in the solution of polymer-free WLMs of potassium oleate after the addition of n-dodecane. As a result, the viscosity *η_0_* of the initial solution decreased to 1.2 mPa·s (Figure 2B).

The decrease in viscosity upon the addition of the hydrocarbon was also observed in the solution of hybrid micelles (Figure 2B). In this case, the viscosity reached a value of 1.5 mPa·s (Figure 2B). The slightly larger value of the resulting viscosity can be explained by the fact that the microemulsion droplets formed in this system are linked to the P4VP chains, as evidenced by the cryo-TEM data (Figure 1D). Similar beads-on-string structures, where a polymer links several micelles, were found in many systems containing various combinations of polymers with ionic surfactants [41,42,43,44]. Thus, the elongated form of the structures in the hybrid system is retained, even in the presence of n-dodecane (Figure 1D). Also, in Figure 1D, one can glean that these structures are oriented in one direction, which may be caused by shear force during blotting used for the preparation of the TEM samples [45,46]. Previously, V. Croce et al. [47] also demonstrated the blotting-driven orientation of WLMs of erucyl bis(hydroxyethyl)methylammonium chloride. This suggests the possible application of P4VP-potassium oleate complexes as DRAs during oil pumping through pipelines, as the energy of the flow will be expended on the alignment of the complexes but not on the formation of turbulence.

**Figure 3:**
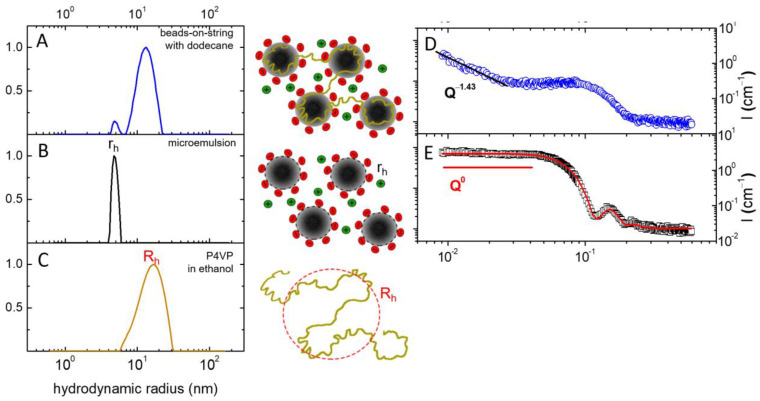
Distributions of hydrodynamic radii in (**A**) solutions of beads-on-string structures formed from hybrid P4VP-potassium oleate micelles upon addition of n-dodecane; (**B**) microemulsion droplets formed from polymer-free potassium oleate micelles upon addition of n-dodecane (black); (**C**) P4VP in ethanol. SAXS profiles (*I* vs. *Q*) of aqueous solutions of beads-on-string structures formed from hybrid P4VP-potassium oleate micelles upon addition of n-dodecane (**D**) and microemulsion droplets formed from polymer-free potassium oleate micelles upon addition of n-dodecane (**E**). Solid line is the fit of the scattering data from microemulsion by form-factor of core-shell sphere. The concentrations of potassium oleate, P4VP, and n-dodecane are 1.5, 0.2, and 1 wt%, respectively. Concentration of KCl: 6 wt%. The straight lines indicate the slopes of the scattering curves in low-*Q* region. Temperature was 20 °C.

DLS and SAXS were used to confirm the formation of beads-on-string structures by the hybrid micelles in the presence of a hydrocarbon. In DLS, the normalized autocorrelation function *g*^(1)^(*q,t*) was observed to decay bi-exponentially (Figure S1A), so fast and slow relaxation modes were observed. The fast mode matches the relaxation mode of the polymer-free microemulsion droplets of n-dodecane stabilized by potassium oleate molecules (Figure S1). The hydrodynamic radius of the microemulsion droplets without the polymer, *r_h_* = 4.6 nm (Figure 3B), is close to that found in [29]. The hydrodynamic radius of the species corresponding to the fast mode in the hybrid system is only slightly higher (*r_h_* = 4.8 nm). Thus, the fast mode can be associated with the diffusion of the microemulsion droplets in the hybrid system. The slow mode, in turn, can be attributed to the diffusion of the polymer chains with bound microemulsion droplets (Figure S1). The corresponding hydrodynamic radius (*R_h_* =13.2 nm) is close to the *R_h_* of P4VP in ethanol (Figure 3C) and coincides with the theoretically calculated *R_h_* for a P4VP coil in a good solvent (see Supplementary Materials for details on the calculation).

SAXS curves *I*(*Q*) of the species formed from hybrid micelles and polymer-free micelles upon the addition of n-dodecane are presented in Figure 3. In the medium-*Q* range, the both scattering curves scale as follows: *I~Q*^0^ (Figure 3D,E); this corresponds to scattering from spherical objects, i.e., microemulsion droplets [29]. The approximation of the scattering curve from the microemulsion by a form-factor of the spherical core of n-dodecane, covered by a shell of potassium oleate, yields a radius of the core and a thickness of the shell equal to 3.9 and 1.9 nm, respectively. Consequently, the total radius of the microemulsion droplets is equal to *r* = 5.8 nm, which is close to that previously obtained in the literature via small-angle neutron scattering (SANS) [29]. The somewhat lower hydrodynamic radius *r_h_* of the droplets detected via DLS may indicate deep penetration of water into the shell of potassium oleate. In the low-Q range, where the scattering from the polymer coil of P4VP contributes appreciably to the SAXS curve, the slope of the curve of the hybrid system changes to −1.43 ± 0.05 (Figure 3D). This slope is characteristic for the coil conformation of P4VP in beads-on-string structures [32]. So, the obtained SAXS results indicate that the shape of the structures is governed by the conformation of a polymer chain, and this finding is consistent with the cryo-TEM and DLS results (Figure 3B,E). Thus, several complementary techniques have shown that the hybrid micelles were transformed into beads-on-string structures representing microemulsion droplets linked to a polymer chain. Such structures should be more effective DRAs for the transport of hydrocarbons than polymer-free WLMs of potassium oleate, as stretching the polymer chains in the flow will induce the dissipation of turbulence kinetic energy [48].

**Figure 4:**
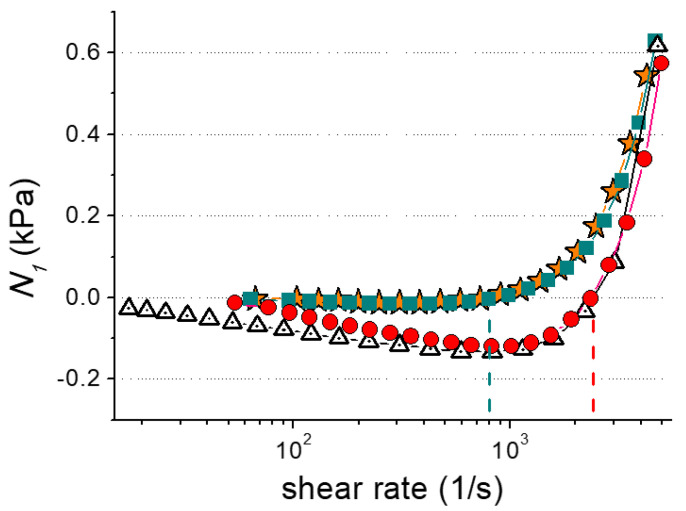
Dependences of first normal stress difference *N*_1_ vs. shear rate of water (stars), aqueous solutions of hybrid P4VP-potassium oleate micelles without hydrocarbon (triangles), beads-on-string structures formed from hybrid P4VP-potassium oleate micelles upon addition of n-dodecane (circles), and microemulsion droplets formed from polymer-free potassium oleate micelles upon addition of n-dodecane (squares). Dashed lines point out critical shear rates, at which turbulence is initiated. The concentrations of potassium oleate, P4VP, and n-dodecane are 1.5, 0.2, and 1 wt%, respectively. Concentration of KCl: 6 wt%.

To verify this assumption in the steady shear rheological experiment, the values of the first normal stress difference *N*_1_, i.e., the normal pressure applied to the cone–plate measuring cell, were determined for three systems: aqueous solutions of hybrid P4VP-potassium oleate micelles without the hydrocarbon, beads-on-string structures formed from hybrid micelles upon the addition of n-dodecane, and polymer-free microemulsion droplets. Pure water was chosen as a reference sample with no drag-reducing efficiency. *N*_1_ is the difference between the two components of the shear stress tensor, namely, *τ*_11_ and *τ*_22_, that are applied perpendicular and parallel to the direction of the flow, respectively [49]. In the cone–plate measuring cell, the shear stress applied to the sample is constant and parallel to the flow [49]. Consequently, at low shear rates that induce laminar flow in the cell, *N*_1_ ≤ 0. At higher shear rates, when turbulent vortices are formed near the surface of the cone, a non-zero value of *τ*_11_ appears, and *N_1_* increases. Thus, the shear rate at which *N*_1_ becomes positive is the critical one and the one that initiates turbulence. As shown by Y. Qi and J.L. Zakin [50,51], the observed method of *N*_1_ evaluation usually correlates with the results of direct drag-reducing-value tests. The dependences of *N*_1_ on shear rate are demonstrated in Figure 4. It can be seen that the *N*_1_ values of the aqueous solution of the polymer-free microemulsion, as well as of pure water, become positive at 800 s^−1^. At the same time, positive *N*_1_ values of the solutions containing hybrid micelles and beads-on-string structures were observed only at 2400 s^−1^ (Figure 4); hence, in the case of pipeline transportation at shear rates less than at 2400 s^−1^, there will be no interaction between the flow and the walls of the tube and, consequently, no turbulence. 

Additionally, in our experiments, the rise in *N*_1_ for all the studied samples was followed by throwing them out from the gap in the measuring cell that resulted in the increase in viscosity (Figure S2). This effect was also attributed in the literature to the formation of turbulent flow [49]. Therefore, a solution of polymer-free WLMs of potassium oleate in the presence of oil possesses very low drag-reducing efficiency comparable to that of pure water. In contrast, the solutions of hybrid micelles, which demonstrated three-fold higher values of critical shear rates (Figure 4), act as effective DRAs, both with and without a hydrocarbon. Thus, H. Sharma and E.E. Dormidontova [32] used computer modelling to show that shear flow breaks hybrid P4VP-potassium oleate micelles into fragments, except for the section armored with polymer, which remained intact. Therefore, the drag-reducing efficiency of hybrid micelles is also governed by embedded polymers. Note that hybrid P4VP-potassium oleate WLMs could be applied as DRAs not only in the process of oil transportation but also while pumping the HF fluid into an oil well.

## 4. Conclusions

Practical applications, wherein surfactant WLMs and polymers are used together, require multi-functional materials that combine the advantages of both components. In the present paper, hybrid WLMs of potassium oleate with embedded P4VP are proposed as an additive to fracturing fluid that permits its application both in oil production and transportation. Several complementary experimental methods (rheology, cryo-TEM, DLS, and SAXS) were applied to investigate their forms and mechanical properties in the presence of hydrocarbon n-dodecane.

According to the rheological data, at shear rates typical for in situ fracturing tests, the aqueous solution of hybrid micelles without hydrocarbon showed a viscosity close to that of the polymer-free WLMs at the same concentration of surfactant. Therefore, it may be used as a thickener for HF fluid similar to a polymer-free surfactant system. The addition of n-dodecane induced a drop in the viscosity of the solution down to 1.5 mPa·s. that, according to cryo-TEM, was attributed to the transformation of hybrid micelles into the beads-on-string structures. Cryo-TEM, DLS, and SAXS confirmed the linking of microemulsion droplets by polymer chains in these structures. As a consequence, they demonstrated higher drag-reducing efficiency compared to polymer-free microemulsion droplets of potassium oleate.

Thus, the observed responsiveness to hydrocarbons permits the hybrid P4VP-potassium oleate micelles to be used in HF without breaker, like in the case of ordinary surfactant-based HF. But, in contrast to surfactant-based fluids, after the absorption of hydrocarbons, the beads in the hybrid system remain linked by polymer chains. The elongated form of beads-on-string structures formed in the hybrid system upon the absorption of hydrocarbon also allows them to be applied as DRAs while transporting the recovered oil through pipelines. 

The described system is a proof of concept that demonstrates the possibility of the concurrent use of hybrid micelles for HF and drag reducing. Their use could provide both a reduction in the cost and the simplification of the sequential procedures of oil recovery and oil transportation. To the best of our knowledge, such a nanomaterial has not been proposed in the literature before. Different compositions of surfactants and polymers could be tested in this regard. 

## Data Availability

The data presented in this study are openly available.

## Supplementary Materials

The following supporting information can be downloaded at https://www.mdpi.com/article/10.3390/polym15234615/s1: Calculation of the hydrodynamic radius for P4VP coil in good solvent; Figure S1: field autocorrelation function *g^(1)^ (q,t)* and the decay time distribution function at scattering angle of θ = 90° for solutions of beads-on-string P4VP-potassium oleate complexes with dodecane, microemulsion droplets of dodecane without polymer, and P4VP in ethanol; Figure S2: dependences of first normal stress difference *N*_1_ and apparent viscosity vs. shear rate of water, hybrid P4VP-potassium oleate micelles without hydrocarbon, aqueous solutions of beads-on-string structures formed from hybrid P4VP-potassium oleate micelles upon addition of n-dodecane, and microemulsion droplets formed from polymer-free potassium oleate micelles upon addition of n-dodecane. Ref. [52] is cited in the Supplementary Materials.

## References

[1] Zana R. (2005). Dynamics of Surfactant Self-Assemblies: Micelles, Microemulsions, Vesicles and Lyotropic Phases.

[2] Israelachvili J.N., Mitchell D.J., Ninham B.W. (1976). Theory of Self-Assembly of Hydrocarbon Amphiphiles into Micelles and Bilayers. J. Chem. Soc. Faraday Trans..

[3] Zhang Q., Shu X.Z., Lucas J.M., Toste F.D., Somorjai G.A., Alivisatos A.P. (2014). Inorganic Micelles as Efficient and Recyclable Micellar Catalysts. Nano Lett..

[4] Jiang Y., Cai Z., Fu S., Gu H., Fu X., Zhu J., Ke Y., Jiang H., Cao W., Wu C. (2023). Relaxivity Enhancement of Hybrid Micelles via Modulation of Water Coordination Numbers for Magnetic Resonance Lymphography. Nano Lett..

[5] Barauskas J., Johnsson M., Tiberg F. (2005). Self-Assembled Lipid Superstructures: Beyond Vesicles and Liposomes. Nano Lett..

[6] Danino D., Talmon Y., Levy H., Beinert G., Zana R. (1995). Branched Threadlike Micelles in an Aqueous Solution of a Trimeric Surfactant. Science.

[7] Landsmann S., Luka M., Polarz S. (2012). Bolaform Surfactants with Polyoxometalate Head Groups and Their Assembly into Ultra-Small Monolayer Membrane Vesicles. Nat. Commun..

[8] Ma S., Hu Y., Wang R. (2015). Self-Assembly of Polymer Tethered Molecular Nanoparticle Shape Amphiphiles in Selective Solvents. Macromolecules.

[9] Zana R., Kaler E.W. (2007). Giant Micelles: Properties and Applications.

[10] Dreiss C.A., Feng Y. (2017). Wormlike Micelles: Advances in Systems, Characterisation and Applications.

[11] Feng Y., Chu Z., Dreiss C.A. (2015). Smart Wormlike Micelles: Design, Characteristics and Applications.

[12] Chu Z., Dreiss C.A., Feng Y. (2013). Smart Wormlike Micelles. Chem. Soc. Rev..

[13] Ezrahi S., Tuval E., Aserin A. (2006). Properties, Main Applications and Perspectives of Worm Micelles. Adv. Colloid Interface Sci..

[14] Berret J.F., Schonbeck N., Gazeau F., El Kharrat D., Sandre O., Vacher A., Airiau M. (2006). Controlled Clustering of Superparamagnetic Nanoparticles Using Block Copolymers: Design of New Contrast Agents for Magnetic Resonance Imaging. J. Am. Chem. Soc..

[15] Wang J., Feng Y., Agrawal N.R., Raghavan S.R. (2017). Wormlike Micelles versus Water-Soluble Polymers as Rheology-Modifiers: Similarities and Differences. Phys. Chem. Chem. Phys..

[16] Moussa W., Colombani O., Benyahia L., Nicolai T., Chassenieux C. (2016). Structure of a Self-Assembled Network Made of Polymeric Worm-like Micelles. Polym. Bull..

[17] Yang J. (2002). Viscoelastic Wormlike Micelles and Their Applications. Curr. Opin. Colloid Interface Sci..

[18] Tong S., Gu M., Singh R., Mohanty K.K. (2019). Proppant Transport in Foam Fracturing Fluid during Hydraulic Fracturing. J. Pet. Sci. Eng..

[19] Philippova O.E., Molchanov V.S. (2019). Enhanced Rheological Properties and Performance of Viscoelastic Surfactant Fluids with Embedded Nanoparticles. Curr. Opin. Colloid Interface Sci..

[20] Tong S., Mohanty K.K. (2016). Proppant Transport Study in Fractures with Intersections. Fuel.

[21] Davoodi S., Al-Shargabi M., Wood D.A., Rukavishnikov V.S. (2023). A Comprehensive Review of Beneficial Applications of Viscoelastic Surfactants in Wellbore Hydraulic Fracturing Fluids. Fuel.

[22] Fan Y., Duan W., Xu K., Yan C., Zheng C. (2023). Zr, N-Co-Doped Carbon Quantum Dot Crosslinking Agents for Use in Fracturing Fluids. ACS Appl. Nano Mater..

[23] Gu Y., Yu S., Mou J., Wu D., Zheng S. (2020). Research Progress on the Collaborative Drag Reduction Effect of Polymers and Surfactants. Materials.

[24] Elbing B.R., Solomon M.J., Perlin M., Dowling D.R., Ceccio S.L. (2011). Flow-Induced Degradation of Drag-Reducing Polymer Solutions within a High-Reynolds-Number Turbulent Boundary Layer. J. Fluid Mech..

[25] Zhao M., Liu S., Dai C., Yan R., Li Y., Liu P. (2023). Development and Drag Reduction Behaviors of a Water-in-Water Emulsion Polymer Drag Reducer. ACS Appl. Polym. Mater..

[26] Barati R., Liang J.T. (2014). A Review of Fracturing Fluid Systems Used for Hydraulic Fracturing of Oil and Gas Wells. J. Appl. Polym. Sci..

[27] Parker A., Fieber W. (2013). Viscoelasticity of Anionic Wormlike Micelles: Effects of Ionic Strength and Small Hydrophobic Molecules. Soft Matter.

[28] Zaldivar G., Conda-Sheridan M., Tagliazucchi M. (2021). Scission Energies of Surfactant Wormlike Micelles Loaded with Nonpolar Additives. J. Colloid Interface Sci..

[29] Shibaev A.V., Tamm M.V., Molchanov V.S., Rogachev A.V., Kuklin A.I., Dormidontova E.E., Philippova O.E. (2014). How a Viscoelastic Solution of Wormlike Micelles Transforms into a Microemulsion upon Absorption of Hydrocarbon: New Insight. Langmuir.

[30] AL-Dogail A., Gajbhiye R., Patil S. (2023). A Review of Drag-Reducing Agents (DRAs) in Petroleum Industry. Arab. J. Sci. Eng..

[31] Kwiatkowski A.L., Sharma H., Molchanov V.S., Orekhov A.S., Vasiliev A.L., Dormidontova E.E., Philippova O.E. (2017). Wormlike Surfactant Micelles with Embedded Polymer Chains. Macromolecules.

[32] Kwiatkowski A.L., Molchanov V.S., Sharma H., Kuklin A.I., Dormidontova E.E., Philippova O.E. (2018). Growth of Wormlike Micelles of Surfactant Induced by Embedded Polymer: Role of Polymer Chain Length. Soft Matter.

[33] Macosco C.W. (1996). Rheology: Principles, Measurements and Applications.

[34] Korchagina E.V., Philippova O.E. (2010). Multichain Aggregates in Dilute Solutions of Associating Polyelectrolyte Keeping a Constant Size at the Increase in the Chain Length of Individual Macromolecules. Biomacromolecules.

[35] SasView. http://www.sasview.org/.

[36] Guinier A., Fournet G. (1955). Small-Angle Scattering of X-rays.

[37] Calabrese M.A., Wagner N.J. (2018). Detecting Branching in Wormlike Micelles via Dynamic Scattering Methods. ACS Macro Lett..

[38] Kwiatkowski A.L., Molchanov V.S., Kuklin A.I., Philippova O.E. (2020). Opposite Effect of Salt on Branched Wormlike Surfactant Micelles with and without Embedded Polymer. J. Mol. Liq..

[39] Calabrese M.A., Rogers S.A., Murphy R.P., Wagner N.J. (2015). The Rheology and Microstructure of Branched Micelles under Shear. J. Rheol..

[40] Croce V., Cosgrove T., Dreiss C.A., King S., Maitland G., Hughes T. (2005). Giant Micellar Worms under Shear: A Rheological Study Using SANS. Langmuir.

[41] Piculell L., Norrman J., Svensson A.V., Lynch I., Bernardes J.S., Loh W. Ionic Surfactants with Polymeric Counterions. Adv. Colloid Interface Sci. 2009, 147–148, 228–236.

[42] Kogej K. (2010). Association and Structure Formation in Oppositely Charged Polyelectrolyte–Surfactant Mixtures. Adv. Colloid Interface Sci..

[43] Langevin D. (2009). Complexation of Oppositely Charged Polyelectrolytes and Surfactants in Aqueous Solutions. A review. Adv. Colloid Interface Sci..

[44] Artykulnyi O.P., Shibaev A.V., Avdeev M.M., Ivankov O.I., Bulavin L.A., Petrenko V.I., Philippova O.E. (2020). Structural Investigations of Poly(Ethylene Glycol)-Dodecylbenzenesulfonic Acid Complexes in Aqueous Solutions. J. Mol. Liq..

[45] Walther A., Müller A.H.E. (2009). Formation of Hydrophobic Bridges between Multicompartment Micelles of Miktoarm Star Terpolymers in Water. Chem. Commun..

[46] Cui H., Hodgdon T.K., Kaler E.W., Abezgauz L., Danino D., Lubovsky M., Talmon Y., Pochan D.J. (2007). Elucidating the Assembled Structure of Amphiphiles in Solution via Cryogenic Transmission Electron Microscopy. Soft Matter.

[47] Croce V., Cosgrove T., Maitland G., Hughes T. (2003). Rheology, Cryogenic Transmission Electron Spectroscopy, and Small-Angle Neutron Scattering of Highly Viscoelastic Wormlike Micellar Solutions. Langmuir.

[48] De Gennes P. (1986). Towards a Scaling Theory of Drag Reduction. Phys. A.

[49] Schramm G.G.S. (1994). A Practical Approach to Rheology and Rheometry.

[50] Qi Y., Littrell K., Thiyagarajan P., Talmon Y., Schmidt J., Lin Z., Zakin J.L. (2009). Small-Angle Neutron Scattering Study of Shearing Effects on Drag-Reducing Surfactant Solutions. J. Colloid Interface Sci..

[51] Qi Y., Kesselman E., Hart D.J., Talmon Y., Mateo A., Zakin J.L. (2011). Comparison of Oleyl and Elaidyl Isomer Surfactant-Counterion Systems in Drag Reduction, Rheological Properties and Nanostructure. J. Colloid Interface Sci..

[52] Grosberg A.Y., Khokhlov A.R. (1994). Statistical Physics of Macromolecules.

