# Supplementary material for: Hybrid Polymer–Surfactant Wormlike Micelles for Concurrent Use for Oil Recovery and Drag Reduction
Source: Polymers (Basel). 2023 Dec 4;15(23):4615. doi: 10.3390/polym15234615 (PMC10708556; doi:10.3390/polym15234615)
Supplement: Supplementary file 1 [file polymers-15-04615-s001.zip › polymers-2739389-supplementary.pdf]

Supporting Information

Hybrid Polymer–Surfactant Wormlike Micelles for Concurrent Use for Oil Recovery and Drag Reduction

Alexander L. Kwiatkowski [1,*], Vyacheslav S. Molchanov [1], Yuri M. Chesnokov [2], Oleksandr I. Ivankov [3] and Olga E. Philippova [1]

[1] Faculty of Physics, Lomonosov Moscow State University, 119991 Moscow, Russia; molchan@polly.phys.msu.ru (V.S.M.); phil@polly.phys.msu.ru (O.E.P.)
[2] National Research Center, Kurchatov Institute, 123182 Moscow, Russia; chessyura@yandex.ru
[3] Joint Institute for Nuclear Research, 141980 Dubna, Russia; ivankov@jinr.ru
* Correspondence: kvyatkovskij@physics.msu.ru

Calculation of hydrodynamic radius R_h of for P4VP coil in good solvent

Hydrodynamic radius of P4VP coil in good solvent R_h might be evaluated, as [1]:

$$R_h = \sqrt{\frac{3\pi}{128}} 2^{2/5} L^{3/5} l_p^{\ 2/5} = 14.2\, nm,$$

where L=542 nm is the averaged contour length of P4VP; l_p = 0.8 nm is its persistence length, previously measured by small-angle neutron scattering in d-DMSO [2].

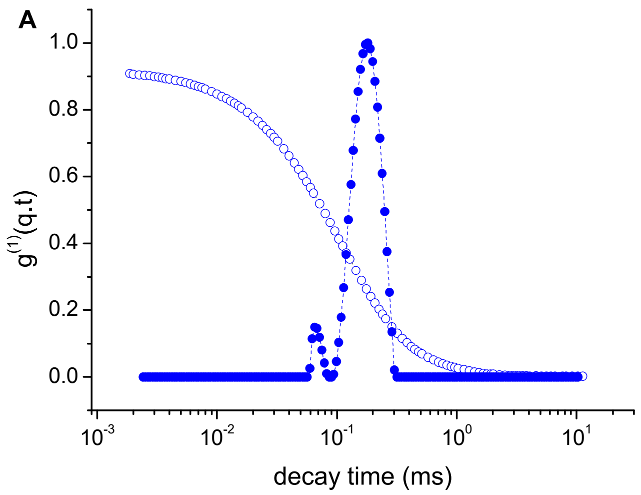

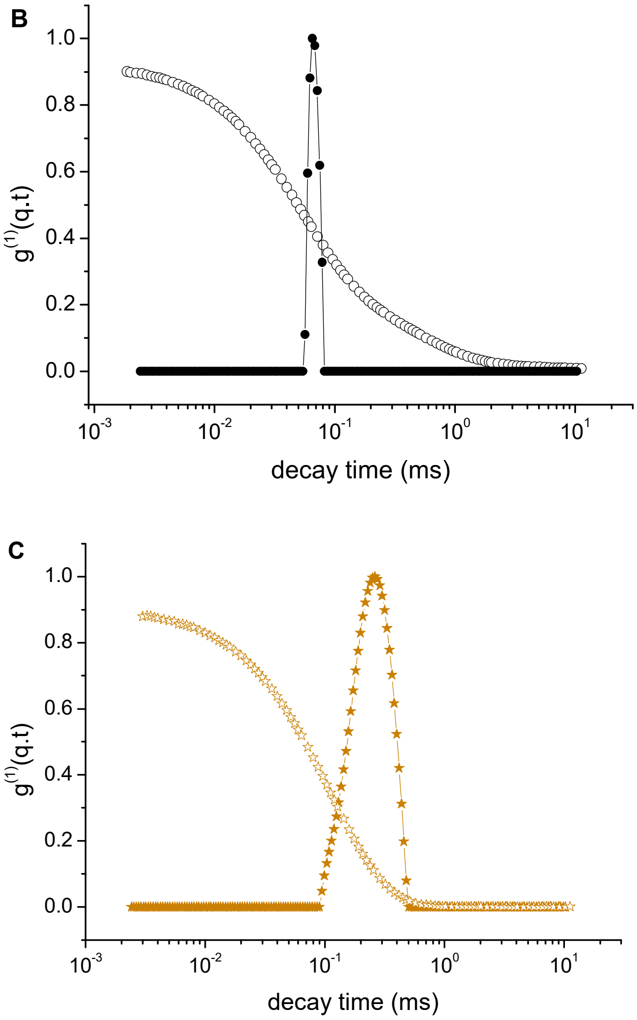

Figure S1. Field autocorrelation function $g^{(1)}$ (q,t) (open symbols) and the decay time distribution function (filled symbols) at scattering angle θ=90° for solutions of beads-on-string P4VP-potassium oleate complexes with dodecane (A); microemulsion drops of dodecane without polymer (B); and P4VP in ethanol (C) at 20°C.

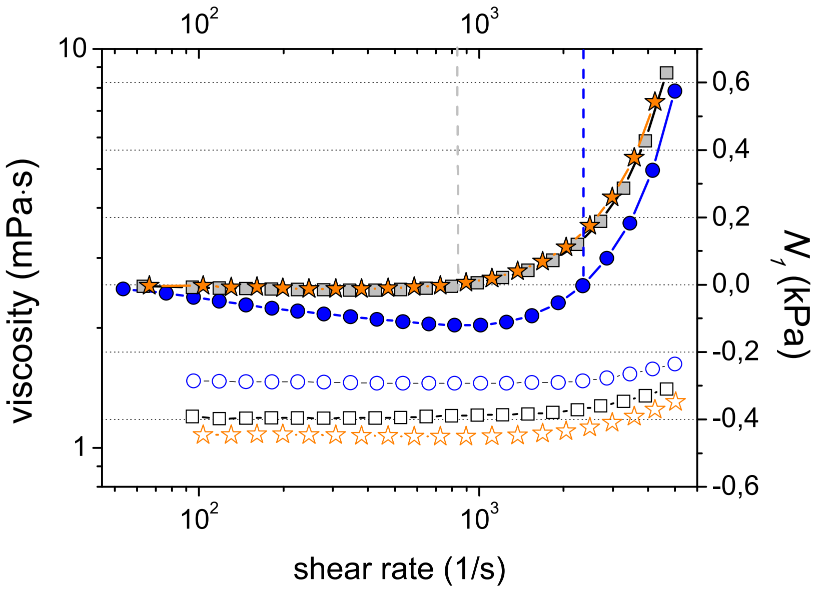

Figure S2. Dependences of first normal stress difference N_1 (filled symbols) and apparent viscosity (empty symbols) vs shear rate of water (stars), hybrid P4VP-potassium oleate micelles without hydrocarbon (triangles), aqueous solutions of beads-on-string structures formed from hybrid P4VP-potassium oleate micelles upon addition of n-dodecane (circles) and microemulsion droplets formed from polymer-free potassium oleate micelles upon addition of n-dodecane (squares). Dashed lines point out on critical shear rates, at which turbulence is induced. Concentrations of potassium oleate, P4VP and n-dodecane are 1.5, 0.2 and 1.0 wt.%, respectively.

References:

1. Grosberg, A.Y.; Khokhlov, A.R. *Statistical Physics of Macromolecules*; American Inst. of Physics, 1994; ISBN 978-1-56396-071-0.
2. Kwiatkowski, A.L.; Molchanov, V.S.; Sharma, H.; Kuklin, A.I.; Dormidontova, E.E.; Philippova, O.E. Growth of Wormlike Micelles of Surfactant Induced by Embedded Polymer: Role of Polymer Chain Length. *Soft Matter* **2018**, *14*, 4792–4804, doi:10.1039/C8SM00776D.
